# Pharmacokinetic interaction of voriconazole and clarithromycin in Pakistani healthy male volunteers: a single dose, randomized, crossover, open-label study

**DOI:** 10.3389/fphar.2023.1134803

**Published:** 2023-06-09

**Authors:** Mehwish Mushtaq, Kshaf Fatima, Aneeqa Ahmad, Osama Mohamed Ibrahim, Muhammad Faheem, Yasar Shah

**Affiliations:** ^1^ Department of Pharmacy, Abdul Wali Khan University Mardan, Mardan, Pakistan; ^2^ Department of Pharmacy, University of Peshawar, Peshawar, Pakistan; ^3^ University Medical and Dental College, The University of Faisalabad, Faisalabad, Pakistan; ^4^ Punjab Medical College, Faisalabad Medical University, Faisalabad, Pakistan; ^5^ College of Pharmacy, University of Sharjah, Sharjah, United Arab Emirates; ^6^ Faculty of Pharmacy, Cairo University, Cairo, Egypt; ^7^ Department of Pharmacy, University of Swabi, Swabi, Pakistan

**Keywords:** voriconazole (voriz), clarithromycin (CLRM), washout period, randomized, pharmacokinetic drug-drug interaction (PK-DDI), clinical significance, crossover, open-label

## Abstract

**Background:** Voriconazole an antifungal drug, has a potential for drug-drug interactions (DDIs) with administered drugs. Clarithromycin is a Cytochromes P450 CYP (3A4 and 2C19) enzyme inhibitor, and voriconazole is a substrate and inhibitor of these two enzymes. Being a substrate of the same enzyme for metabolism and transport, the chemical nature and pKa of both interacting drugs make these drugs better candidates for potential pharmacokinetic drug-drug interactions (PK-DDIs). This study aimed to evaluate the effect of clarithromycin on the pharmacokinetic profile of voriconazole in healthy volunteers.

**Methods:** A single oral dose, open-label, randomized, crossover study was designed for assessing PK-DDI in healthy volunteers, consisting of 2 weeks washout period. Voriconazole, either alone (2 mg × 200 mg, tablet, P/O) or along with clarithromycin (voriconazole 2 mg × 200 mg, tablet + clarithromycin 500 mg, tablet, P/O), was administered to enrolled volunteers in two sequences. The blood samples (approximately 3 cc) were collected from volunteers for up to 24 h. Plasma concentrations of voriconazole were analyzed by an isocratic, reversed-phase high-performance-liquid chromatography ultraviolet-visible detector (RP HPLC UV-Vis) and a non-compartmental method.

**Results:** In the present study, when voriconazole was administered with clarithromycin versus administered alone, a significant increase in peak plasma concentration (Cmax) of voriconazole by 52% (geometric mean ratio GMR: 1.52; 90% CI 1.04, 1.55; *p* = 0.000) was observed. Similarly, the area under the curve from time zero to infinity (AUC^0-∞^) and the area under the concentration-time curve from time zero to time-t (AUC^0-t^) of voriconazole also significantly increased by 21% (GMR: 1.14; 90% CI 9.09, 10.02; *p* = 0.013), and 16% (GMR: 1.15; 90% CI 8.08, 10.02; *p* = 0.007), respectively. In addition, the results also showed a reduction in the apparent volume of distribution (Vd) by 23% (GMR: 0.76; 90% CI 5.00, 6.20; *p* = 0.051), and apparent clearance (CL) by 13% (GMR: 0.87; 90% CI 41.95, 45.73; *p* = 0.019) of voriconazole.

**Conclusion:** The alterations in PK parameters of voriconazole after concomitant administration of clarithromycin are of clinical significance. Therefore, adjustments in dosage regimens are warranted. In addition, extreme caution and therapeutic drug monitoring are necessary while co-prescribing both drugs.

**Clinical Trial Registration:** clinicalTrials.gov, Identifier NCT05380245.

## 1 Introduction

Drug-drug interactions (DDI) occur when one drug (perpetrator drug) varies the plasma concentration and the biological outcomes of a drug (victim drug) (Hasnain et al., 2017). There are two types of DDIs, i.e., Pharmacokinetic drug-drug interactions (PK-DDIs) and Pharmacodynamic drug-drug interactions (PD-DDIs). PK-DDIs result from changes in plasma concentrations of a ‘victim’ drug caused by a ‘perpetrator’ drug altering the metabolism or transporter-mediated disposition of the victim drug. In particular, the cytochrome P450 (CYP) system, which is responsible for the metabolism of many drugs, can be influenced by other drugs leading to PK-DDIs. Induction of CYP enzymes can increase the metabolism and clearance of a victim drug, resulting in reduced plasma concentrations and potentially reduced efficacy. On the other hand, inhibition of CYP enzymes can decrease the metabolism and clearance of a victim drug, leading to increased plasma concentrations and potentially increased risk of adverse effects (Storelli et al., 2018). Drug transporters, such as P-glycoprotein (P-gp), multidrug resistance protein 2 (MRP2), and breast cancer resistance protein (BCRP), also play a significant role in drug absorption and excretion. Inhibition or induction of these transporters can affect the bioavailability and elimination of drugs, leading to PK-DDIs (Marchetti et al., 2007; Niwa and Hata, 2016). Other factors, such as age, gender, nutritional status, diseases, genetic polymorphisms, and ontogeny of metabolic enzymes, can also impact drug metabolism and contribute to PK-DDIs. For example, some drugs may have different pharmacokinetic profiles in elderly patients than in younger individuals due to age-related changes in drug metabolism. Understanding and predicting PK-DDIs are crucial in clinical practice to optimize medication therapy and prevent adverse effects. Healthcare professionals should be vigilant in considering potential interactions when prescribing or adjusting drug regimens, and patients should inform their healthcare providers about all the medications they are taking, including prescription, over-the-counter, and herbal products, to minimize the risk of PK-DDIs. Pharmacokinetic drug-drug interactions can be managed through appropriate drug selection, dosing adjustments, and close monitoring of drug concentrations and clinical response. In some cases, alternative medications with lower interaction potential may be chosen, or the timing of drug administration may be adjusted to minimize the risk of PK-DDIs. Overall, pharmacokinetic drug-drug interactions can significantly impact the safety and efficacy of medications by altering their absorption, distribution, metabolism, or excretion. Therefore, understanding the mechanisms and factors contributing to PK-DDIs is critical for healthcare professionals to make informed decisions in medication management, optimize patient outcomes, and minimize the risk of harm (Marchetti et al., 2007; Niwa and Hata, 2016; Hasnain et al., 2017; Storelli et al., 2018). Similarly, our study drug (Voriconazole) is a narrow therapeutic index drug; requiring close monitoring when administered with other drugs (Ashbee et al., 2013). Therefore, it is essential to characterize the PK-DDIs potential of Voriconazole with co-administered drugs.

Voriconazole synthetically derived from fluconazole antifungal agent (Wong‐Beringer and Kriengkauykiat, 2003), having a chemical composition [(2R, 3S) -2- (2, 4-difluorophenyl) -3-(5-fluora-4pyrimidinyl) -1- (1H −1, 2, 4-trizole-1-yl) -2-butanol] and has a broad spectrum (Greer, 2003; Herbrecht, 2004). Voriconazole is rapidly absorbed and has 96% oral bioavailability (B.A) (Geist et al., 2013; Hohmann et al., 2016). Voriconazole is highly metabolized by the hepatic enzyme CYP2C19 and forms a voriconazole-N-oxide as a major inactive metabolite; other metabolites formed are hydroxyl voriconazole and dihydroxy-voriconazole (Greer, 2003). Voriconazole shows the first-pass effect by primary systemic metabolism occurring by cytochrome-P450 enzymes, for example, CYP2C19, CYP2C9, CYP3A4, and CYP3A5. Up to 25% of metabolism occurs by Flavin containing mono-oxygenase FMO-1 and FMO-3 in enterocytes and hepatocytes (Yanni et al., 2008; Vanhove et al., 2017). Voriconazole is a potent inhibitor of CYP2C19, CYP2C9, CYP2B6, and CYP3A4 of hepatocytes and enterocyte enzymes (Jeong et al., 2009). Moreover, voriconazole is administered (oral or IV); its total dose has been excreted as metabolites (98%) within 48 h (Roffey et al., 2003). Renal and Biliary excretion of voriconazole (the metabolized form) is about 75%–80% and 20%–25%, respectively, while the remaining 2% is excreted in the urine in an unchanged form (15). Deliberating voriconazole pharmacokinetics and considerable inter-individual variability in drug disposition have been reported because, in drug disposition, genetic polymorphism of the metabolizing enzymes may have a starring role (Levêque et al., 2006; Hohmann et al., 2016). Voriconazole is also a substrate of p-glycoprotein (ABCB1) located at different sites (intestines and excretory organs) (Mikus et al., 2011). Allegra et al. reported that breast cancer resistance protein (BCRP1), multidrug resistance-associated protein (MRP2, also known as ABCC2), ABCG2, and solute carrier organic anion transporter (SLCO1B3, also known as OATP1B3) transporters might have a role in variation in voriconazole plasma-concentration in pediatrics (Allegra et al., 2018). Voriconazole is an inhibitor of several transporters like BCRP, p-glycoprotein, MRP (its other members MRP-1, MRP-2, MRP-4, and MRP-5), and bile salt export pump (BSEP) (Lempers et al., 2016).

Clarithromycin (6-O-Methylerthromycin) is a semi-synthetic macrolide antibacterial agent with a 14-membered ring (Alkhalidi et al., 2008). Clarithromycin is a frequently prescribed antibiotic drug nowadays. Clarithromycin is a substrate of several transporters (ABCB1, ABCC2, OATP2B1, and OATP1A2) located at different sites (intestinal, hepatic, and renal) (Peters et al., 2011). Clarithromycin is also an inhibitor of p-glycoprotein located at enterocytes (luminal), hepatocytes (canalicular), and renal (luminal) sites, as well as an inhibitor of OATP1B1 and OATP1B3 located at hepatocytes (sinusoidal) and intestine (Wakasugi et al., 1998; Niemi, 2007; Müller and Fromm, 2011). Clarithromycin is extensively metabolized by hepatic CYP3A4. Clarithromycin is an intense inhibitor of CYP3A4 and has a moderate inhibitory activity of CYP2C19, CYP2D6, and CYP1A2 enzymes present at the hepatic and intestinal level (Michalets, 1998; Furuta et al., 1999). Clarithromycin is a recognized inhibitor of CYP3A4, while many drugs are a substrate of this enzyme, so clarithromycin alters the AUC and plasma concentration of astemizole (Rodvold, 1999), cisapride (Haarst et al., 1998) and pimozide (Desta et al., 1999). As a result of PK-DDI, clarithromycin raises the AUC of these drugs (Michalets, 1998; Rodvold, 1999).

Clarithromycin is weakly basic in nature (Grübel and Cave, 1998), with 8.76 PKa (Nakagawa et al., 1992). Voriconazole exhibits a set of pKa values, i.e., basic-1.76 PKa value (Adams and Bergold, 2005; Adams et al., 2008) and acidic PKa values: 4.36 and 12.7 (Owens et al., 2000; Damle et al., 2011; Vanstraelen et al., 2015), respectively. In this viewpoint, the chemical nature as evident by pKa of both interacting drugs (voriconazole and clarithromycin co-administered simultaneously) make them candidates for possible potential PK-DDIs. Likewise, clarithromycin and voriconazole have 42%–72% (Langtry and Brogden, 1997) and 58% (Geist et al., 2013) protein binding, respectively. Clarithromycin is CYP3A4 (Gorski et al., 1998) and CYP2C19 (Furuta et al., 1999) enzyme inhibitor, and voriconazole is also a substrate (Vanhove et al., 2017) and inhibitor (Jeong et al., 2009) of these two enzymes; hence both candidate drugs share the same enzyme pathway. Being a substrate of the same enzyme and transporter, there is a likelihood of PK-DDI between voriconazole and clarithromycin. Enzyme CYP2C19 has genetic polymorphism making the population fall as poor, moderate, and extensive metabolizers (Bahar et al., 2017). Asian peoples are mostly poor CYP2C19 metabolizers, so that DDI may be possible in this region, and voriconazole may show variable C_max_ because of non-linearity (Mikus et al., 2011). Previously reported patterns of voriconazole-DDIs (Donnelly and De Pauw, 2004; Pasqualotto et al., 2010; Dolton et al., 2014; Bahar et al., 2017) and clarithromycin-DDIs (Michalets, 1998; Rodvold, 1999), as well as the PK parameter of both drugs, predicted that DDI might be possible. There is a possibility of co-administration of both drugs in certain clinical situations (Purkins et al., 2003a; Soler-Palacín et al., 2012; Mishima et al., 2017; Hirai et al., 2022). Therefore, we aimed to evaluate the interaction between voriconazole and clarithromycin in healthy Pakistani male volunteers. Till date, no study has been reported on assessing the effect of clarithromycin on the pharmacokinetic parameters of voriconazole.

## 2 Materials and methods

### 2.1 Study objective

The main objectives of this study were to evaluate the pharmacokinetic drug-drug interaction of voriconazole with clarithromycin and its impact on the pharmacokinetic parameters of voriconazole.

### 2.2 Ethical approval

**FIGURE 1:**
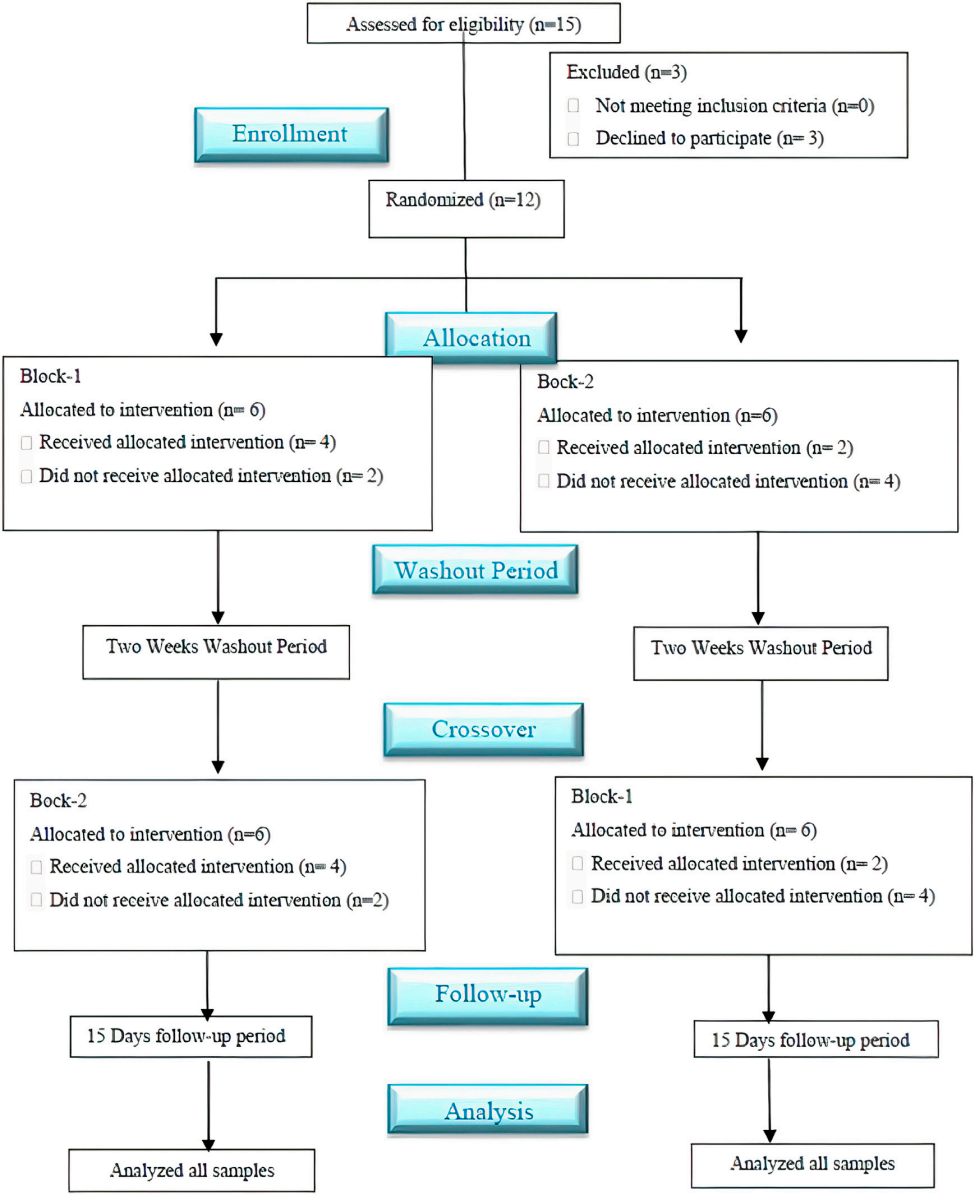
Schematic flow diagram of the clinical study followed the CONSORT guidelines. *A single-dose, randomized, crossover, open-labeled, and two-sequence study with a two-week washout period evaluated the impact of clarithromycin on the pharmacokinetics (PK) profile of voriconazole Pakistani healthy male volunteers.

The study was conducted in the medical dispensary of Abdul Wali Khan University Mardan, Pakistan. The ethical approval was taken from the Advanced Studies and Research Board (ASRB) of the Pharmacy department, Abdul Wali Khan University, Mardan, Pakistan, before the initiation of the study. The study followed “ethical principles of the Helsinki declaration for medical research involving human subjects” and “good clinical practice guidelines.” The clinical trial of this study followed the guidelines of CONSORT (Schulz et al., 2010) (Figure 1).

The entire protocol of this study was published on the clinicalTrial.gov registry as the reference number (ClinicalTrials.gov Identifier: NCT05380245, Additional file: 1). All steps from drug administration to sampling were explained to all enrolled volunteers then they voluntarily signed the informed consent (Additional file: 2).

### 2.3 Trial population

Pakistani twelve male volunteers (*n* = 12) in good health, aged 20–35 years; weight 60–73 (kg); height 1.62–1.79 (m); body mass index (BMI) 22.50–24.90 (kg/m^2^) (according to Quetelet’s index) were enrolled as participants in this PK-DDI study. The selection was based on a detailed medical history, clinical examination, and drug screening in urine. Further, the voriconazole hypersensitivity test and various biochemical tests were also conducted. Volunteers with a history of deviation from normal values in a biochemical test report were excluded from the study. Volunteers who were allergic to both interacting drugs (voriconazole or clarithromycin) were excluded from the study. In addition, those participants who had any clinically significant pathology like chronic renal disease, hepatic impairment, gastrointestinal tract (GIT) allergies or disease (that affected the drug absorption), and hematopoietic illness were also excluded from the study. Half-month before initiation and during the clinical trial, the included volunteers were restricted from smoking, caffeine, and taking any pharmaceutical or herbal medication other than candidate drugs (study period only). The study participants were not allowed to take grapefruit juice continuously for 2 weeks before the study and till the termination of a clinical trial (Sugar and Liu, 2000). Written consent was obtained from all included volunteers in the PK-DDI study. Alcohol or snuff addicted, smokers, caffeine or methylxanthine consumer, and volunteers who did not sign the permission/consent form were excluded from the study.

### 2.4 Study design

The study designed was a single oral dose, open-labeled, randomized, crossover, and consisted of 02 weeks of washouts in between for evaluating drug-drug interaction in healthy volunteers. Voriconazole, either alone (2 mg × 200 mg, tab, P/O) or in combination with clarithromycin (voriconazole 2 × 200 mg, tab + clarithromycin 500 mg, tab, P/O), was administered to enrolled participants in two sequences. The product information is given in Table 1 whereas, the study design is shown in Table 2.

**TABLE 1:** Reference and interacting-formulations used in PK-DDI of Voriconazole.

Reference formulation	Intervention/Test formulation
Tablets Vfend^*®*^, 200 mg by Pfizer, Inc. (United States)	Tablets Klaricid^*®*^, 500 mg by Abbott, Lab Pvt. Ltd. (Karachi, Pakistan)	
Batch No: 00005505; Mfg. Date September 2016	Batch No: 81573XU; Mfg. Date October 2017	

*Vfend^®^, voriconazole; klaricid^®^, clarithromycin.

**TABLE 2:** Study design for the pharmacokinetic DDI-study of voriconazole with clarithromycin.

Block	Random	Volunteer number #	Treatment sequence-I	Washout period	Treatment sequence-II
	Code #				
B1	0.1741103	1	Voriz (Alone)*	*Two Weeks Washout Period (Reduction of Carry-Over Effect)*	Voriz + CLRM*
B2	0.2111928	2	Voriz (Alone*		Voriz + CLRM*
B1	0.5978181	3	Voriz + CLRM*		Voriz (Alone)*
B1	0.4155855	4	Voriz + CLRM*		Voriz (Alone)*
B2	0.4991418	5	Voriz (Alone)*		Voriz + CLRM*
B1	0.3008633	6	Voriz + CLRM*		Voriz (Alone)*
B2	0.3427233	7	Voriz (Alone)*		Voriz + CLRM*
B2	0.0239982	8	Voriz + CLRM*		Voriz (Alone)*
B1	0.3583639	9	Voriz + CLRM*		Voriz (Alone)*
B2	0.7956311	10	Voriz (Alone)*		Voriz + CLRM*
B2	0.5435984	11	Voriz + CLRM*		Voriz (Alone)*
B1	0.8531566	12	Voriz (Alone)*		Voriz + CLRM*

Voriz (Alone)* _→_ Dose of 200 mg × 2 mg tablets of voriconazole only.

Voriz + CLRM* →Dose 200 mg × 2 mg tablets of voriconazole + 500 mg × 1 mg tablets of clarithromycin.

### 2.5 Randomization and drug administration

Enrolled volunteers were divided randomly into block-1 and block-2 by the “permuted block randomization” technique, shown in Table 2. By computing the volunteer data into an excel sheet and applying a RAND* function, a two-block (B-1 and B-2) size was selected. Finally, six participants were assigned to each study block for two (Voriz (alone) or Voriz + CLRM) interventions arm for the execution of block randomization. This randomization technique provided a balance (1:1) across both intervention arms. Treatment drugs were administered to enrolled volunteers in two sequences,

Sequence-I: In the first phase, block-1 volunteers on day 1 received oral voriconazole (2 mg × 200 mg, tab, P/O) only. In comparison, block-2 volunteers received oral clarithromycin (500 mg, tab, P/O) along with voriconazole (2 mg × 200 mg, tab, P/O). A 2-week washout period was allocated from day 2 to day 15 to avoid the carry-over effect. Sequence-II: On day 16, the second phase of the trial was conducted, in which block-1 volunteers received voriconazole (2 × 200 mg, tab, P/O) along with clarithromycin (500 mg, tab, P/O), while block-2 volunteers received voriconazole (2 mg × 200 mg, tab, P/O) only. Voriconazole and clarithromycin were administered to the overnight fasted volunteers corresponding to the sequences in Table 1. All volunteers took the medication with a glass of water (250 mL). On days 1 and 16 (treatment days), two and 6 hours after drug administration, standard breakfast and lunch were served to all volunteers, respectively.

### 2.6 Sample collection

The blood samples (approximately 3 cc) were collected from both block-1 and block-2 volunteers in heparinized tubes at specific time points of 0.0 (per dose), 0.5, 1.0, 1.5, 2.0, 2.5, 3.0, 4.0, 6.0, 8.0 and 24 h after administration of two tablets of voriconazole (200 mg, P/O) either alone or co-administration with clarithromycin (500 mg, one tab, P/O). After taking blood samples, immediately these samples were centrifuged (at 500 rpm for 10–15 min) to separate plasma from blood (RBC) and stored at −80°C till analysis.

### 2.7 Safety monitoring of volunteers

After the second sequence of drug administration, follow-up data were collected for 2 weeks (from day 16 to day 30) from all volunteers regarding any side effects or toxicity-related issues. Blurred vision was observed in two volunteers that persisted only for 10–15 min and then subsided. One of our volunteers had felt dizziness during our clinical trials as voriconazole is a narrow therapeutic index drug and also one of the cumulative incidence of adverse events related to neurotoxicity according to version 4.0 of the Common Terminology Criteria for Adverse Events (CTCAE) is dizziness (Jang et al., 2005; Zonios et al., 2008; Ashbee et al., 2013; Zrenner et al., 2014; Bayhan et al., 2016). Blurring vision has been reported as a major side effect in the literature (Theuretzbacher et al., 2006). Voriconazole’s normal therapeutic range in human plasma is 1–5 μg/mL (Boyd et al., 2012), whereas the C_max_ value of one of our volunteers was 5 μg/mL observed. In the follow-up period, we carefully monitored the aspartate aminotransferase (AST) and alanine transaminase (ALT) biochemical test reports of that volunteer. AST and ALT test values slightly increased and then returned to normal in a week.

### 2.8 Sample analysis for determination of voriconazole

An isocratic, reversed-phase high-performance-liquid chromatography ultraviolet-visible detector (RP HPLC UV-Vis) method was initially developed for the analysis of voriconazole standard (stock-solution) and in plasma samples (Mushtaq et al., 2022). Frozen samples of voriconazole and voriconazole + clarithromycin were brought back to working condition at room temperature by thawing in the palms technique. The plasma samples were subjected to protein precipitation and drug extraction with acetonitrile (ACN). A fixed volume of plasma (200 μL), ACN (200 μL), and internal standard (i.e., 2 μg/mL of fluconazole), taken into Eppendorf tubes were vortexed (for 5 min) and kept in the centrifuge at 10,000 RPM for 10–15 min for protein separation. Their supernatant (organic layer) layer was cautiously separated and analyzed by the already developed method. Chromatographic conditions of the HPLC-UV method comprised of isocratic mobile-phase ACN: H_2_O in 60:40 v/v proportions at a flow rate of 1.5 mL/min and UV detection at 254 nm. Then each sample was analyzed at least three times using the Flexar-series HPLC system, Norwalk, USA, by utilizing a C-18 Perkin-Elmer^®^ column (with particulars of 150 mm length, 4.6 mm inner diameter, and 5 μm particle size). The total run time for each sample was ≤7.0 min. The peak of voriconazole and fluconazole (internal standard) were visible at 5.25 and 4.20 min retention time, respectively. The correlation coefficient for voriconazole was observed to be 0.999. The average recovery (in percent) of voriconazole was 97.4%, while the % relative standard deviation (RSD) value was ≤2%. The lower limit of detection was 0.01 μg/mL, whereas, lower limit of quantification was 0.03 μg/mL, respectively. The results expressed that the adapted method of voriconazole has high recovery (Mushtaq et al., 2022).

### 2.9 Pharmacokinetic evaluation

The pharmacokinetic (PK) parameters used for PK-DDI assessment and plasma drug concentration vs. time profile were analyzed statistically through a non-compartmental approach.Pharmacokinetics PK-Summit^®^ (version 2.0.2; Summit Research Services, Ashland, OH) software was used to evaluate all pharmacokinetic parameters. The various non-compartmental pharmacokinetic (PK) parameters calculated were peak plasma concentration (Cmax, µg/mL), time to reach Cmax (tmax, h), the elimination half-life (E-t_1/2_, h), an area under the curve from time zero to infinity (AUC^0-∞^, μg×h/mL), and the area under the concentration-time curve from time zero to time-t (AUC^0-t^, μg×h/mL), mean residence time (MRT, h), elimination rate (Erate, 1/h), apparent clearance (CL/Kg, L/h/Kg) and apparent volume of distribution (Vd, L/Kg).

### 2.10 Statistical data interpretation

A sample of 12 subjects was considered sufficient to detect a difference of 0.2 (20%) AUC^0−t^ in with probability 0.8 when testing (two-sided) at the 5% level (Purkins et al., 2003b). Descriptive statistical tests were performed using SPSS software (version 21.0; IBM Crop; SPSS^®^; 2012); for a non-compartmental approach Pharmacokinetics PK-Summit^®^ (version 2.0.2; Summit Research Services, Ashland, OH) software and MS-Excel used for results evaluation, and such data were presented graphically. The geometric mean ratios were constructed on the geometric mean of voriconazole alone and co-administered voriconazole with clarithromycin for all PK parameters of voriconazole except tmax. A *p* < 0.05 value was considered statistically significant for two tail tests where 90% confidence intervals (CIs) of log-transformed PK parameters were constructed on the estimated marginal means using linear mixed-effects for both treatment groups (voriconazole alone and co-administered voriconazole with clarithromycin). The SPSS software (version 21.0; IBM Crop; SPSS^®^; 2012) procedure MIXED was used with treatment and visits as a fixed effect and subject as a random effect using the Residual maximum likelihood REML method. Sharpiro-Wilk test was used to check the normality of PK parameters. Log transformation was applied to those PK parameters (such as C_max_, MRT, apparent Vd, and E-t_1/2_) which were not normally distributed. Adjusted mean treatment differences in all PK parameters of voriconazole, along with their corresponding confidence intervals (CIs), were estimated from the model. These differences were evaluated by the ratios of geometric means between treatments and used a 90% CI for these ratios. After administration of voriconazole alone and co-administered voriconazole with clarithromycin, the difference between all PK parameters of voriconazole was reported in percentages by exercising this equation:

$$PK\;parameter\;Difference\;in\;\%=\left(b-a\right)/a*100$$

Where;

a: Any PK parameter value of voriconazole after administration of Voriz alone.

b: Any PK parameter value of voriconazole after administration of Voriz + CLRM.

## 3 Results

**FIGURE 2:**
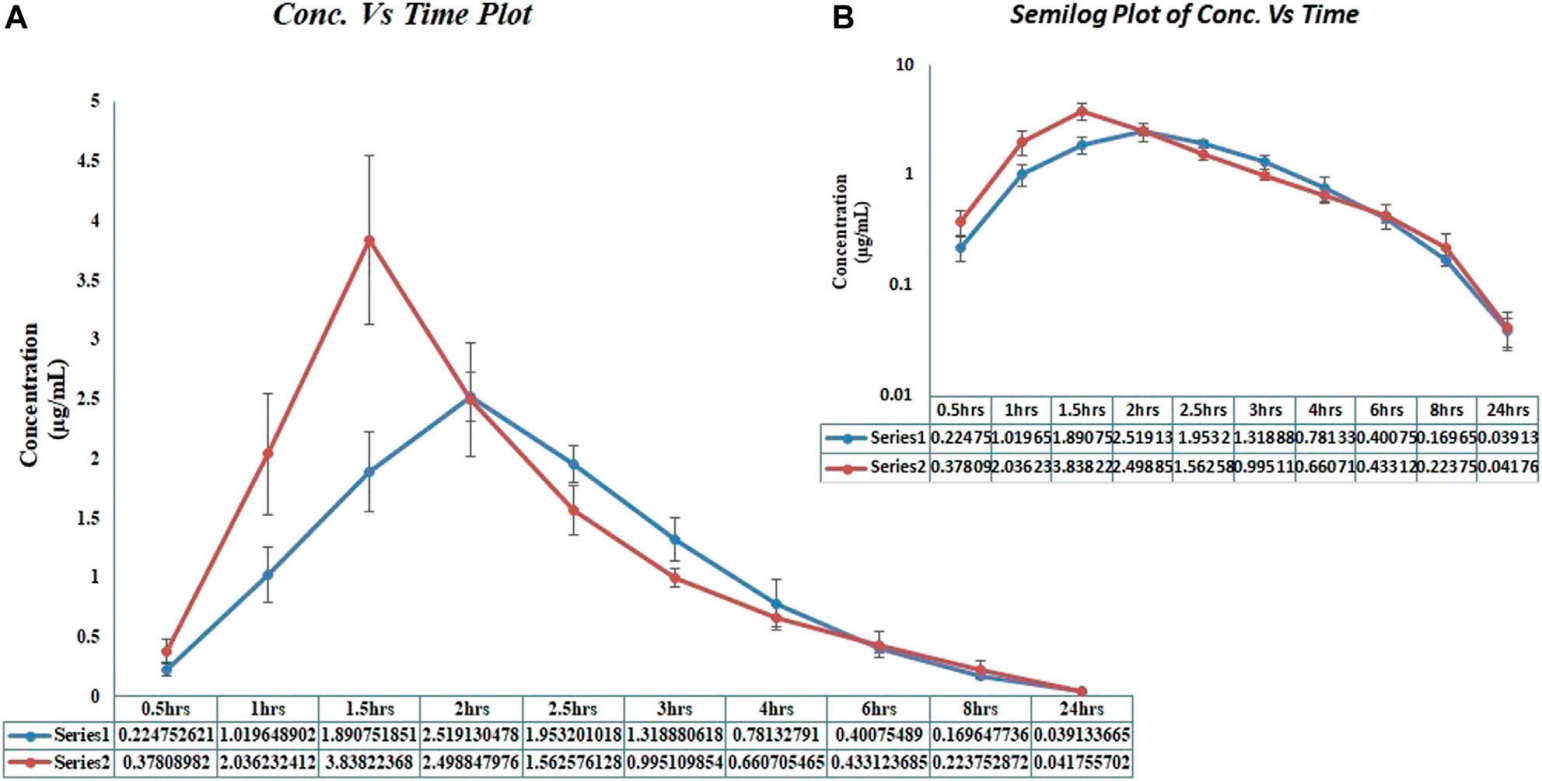
Voriconazole plasma concentration vs. time profiles in healthy male volunteers, after administration of voriconazole alone and after administration of voriconazole along with clarithromycin. *(A) Linear graph. **(B)** Semi-log graph;*Series 1 and blue coloured curves in the graph represented voriconazole concentration, after voriconazole 400 mg administration alone;*Series 2 and red coloured curves in the graph represented voriconazole concentration, after voriconazole 400 mg administration along with clarithromycin 500 mg.

In the current PK-DDI study of voriconazole with clarithromycin, we have enrolled more than 20-year aged healthy Pakistani male volunteers (*n* = 15). However, three out of these 15 volunteers later withdrew due to personal problems. Therefore, the DDI study was carried out on the remaining 12 volunteers (as presented in Figure 1), and these 12 subjects were selected according to mentioned criteria. Furthermore, the range of volunteers’ age, along with their mean with standard deviation (±SD), was 21–25 years and 23.3 ± 1.23 years, respectively, while the range of volunteer’s weight, height, and BMI, as well as their mean with ±SD, was 63–71 kg, 1.62–1.79 m, and 22.50–24.90 kg/m^2^ and 67.51 ± 2.47 kg, 1.69 ± 0.04 m and 23.77 ± 0.91 kg/m^2^, respectively. In addition, an isocratic, reversed-phase high-performance liquid chromatography ultraviolet/visible detector (RP HPLC UV-Vis) method was developed to analyze the voriconazole standard (stock-solution) and voriconazole in plasma samples. The method offered a simple liquid–liquid extraction LLE technique, which exhibited best recovery of voriconazole along with fluconazole, i.e., internal standard. Different experimental conditions were tried and ultimately, the best outcomes were accomplished utilizing C-18 Perkin-Elmer^®^ column with particulars of 150 mm length, 4.6 mm inner diameter and 5 μm particle size, utilizing mobile-phase of acetonitrile-water (ACN: H_2_O) in a proportion of 60: 40 v/v, having a flow rate of 1.5 mL/min, and wavelength of 254 nm. All the analytes were observed to be separated in ≤7 min. The peak of voriconazole and fluconazole (internal standard) were visible at 5.25 and 4.20 min retention time, respectively. The correlation coefficient of voriconazole was observed to be 0.999, and average recovery (in percent) was 97.4%, whereas the relative standard deviation value was ≤2%. The lower limit of detection LLOD was 0.01 μg/mL, whereas lower limit of quantification LLOQ was 0.03 μg/mL, respectively. The results expressed that the adapted method of voriconazole has high recovery (Mushtaq et al., 2022). Further, semi-log and linear graphs of plasma concentrations of voriconazole were plotted as a function of time after administration of voriconazole alone and voriconazole along with clarithromycin, as graphically represented in Figure 2A and 2B. PK-Summit^®^ (version 2.0.2; PK Solutions) SPSS software (version 21.0; IBM Crop; SPSS^®^; 2012), and Microsoft Excel were used to calculate mean with standard deviations, % difference, geometric mean ratio and confidence interval for all PK parameters of voriconazole, as summarized in Table 3.

**TABLE 3:** Comparative pharmacokinetics of voriconazole after administration of voriconazole alone and concomitant administration with clarithromycin.

PK parameters of voriconazole	Mean & std. Deviation voriz (Alone)*	Mean &std. Deviation voriz + CLRM*	% difference	Geometric mean ratio	90% confidence interval (CI)	*p*-value
C_maax_ (µg/mL)	2.52 ± 0.21	3.84 ± 0.562	52%	1.52	(1.04, 1.55)	0.000*
AUC^0-t^ (µg × h/mL)	8.6 ± 0.72	10.02 ± 0.60	16%	1.15	(8.08, 10.02)	0.007*
AUC^0-∞^ (µg × h/mL)	9.09 ± 0.79	11.02 ± 1.09	21%	1.14	(9.09, 11.24)	0.013*
Apparent CL/kg (L/h/kg)	46.77 ± 3.91	40.91 ± 7.16	−13%	0.87	(41.95, 45.73)	0.019*
Apparent Vd/kg (L/kg)	530.85 ± 156.23	411.36 ± 166.72	−23%	0.76	(5.00, 6.20)	0.051
E Half-life (h)	7.94 ± 0.53	5.91 ± 0.71	−26%	0.88	(1.88, 2.05)	0.371
MRT (h)	6.16 ± 0.95	5.55 ± 0.36	−10%	0.89	(1.69, 1.82)	0.321
E_rate_ (1/h)	0.09 ± 0.02	0.11 ± 0.02	12%	1.14	(0.09, 0.11)	0.44
t_max_ (h)	2.00a ± 0	1.50a ± 0	−25%	_	_	_

*As per the linear mixed model, log-transformed C_max_, AUC^0–∞^, and apparent CL, are statistically significant; as per the linear mixed model, log-transformed AUC^0–t^, E Half-life, Apparent Vd, E_rate_, and MRT, are not statistically significant., * shows statistical significance *p* = <0.05; a. The geometric mean ratio GMR, and CI, cannot be computed because the standard error of the difference is 0; Voriz (Alone)* → Dose of 200 mg × 2 mg tablets of voriconazole only; Voriz + CLRM* →Dose 200 mg × 2 mg tablets of voriconazole + 500 mg × 1 mg tablets of clarithromycin; C_max_, maximum plasma concentration; t_max_, time to reach C_max_; MRT, mean residence time; AUC, area under the curve; Cl, clearance; Vd, volume of distribution, and E Half-life t_1/2_, elimination half-life; E_rate_, elimination rate.

### 3.1 PK parameters of voriconazole

**FIGURE 3:**
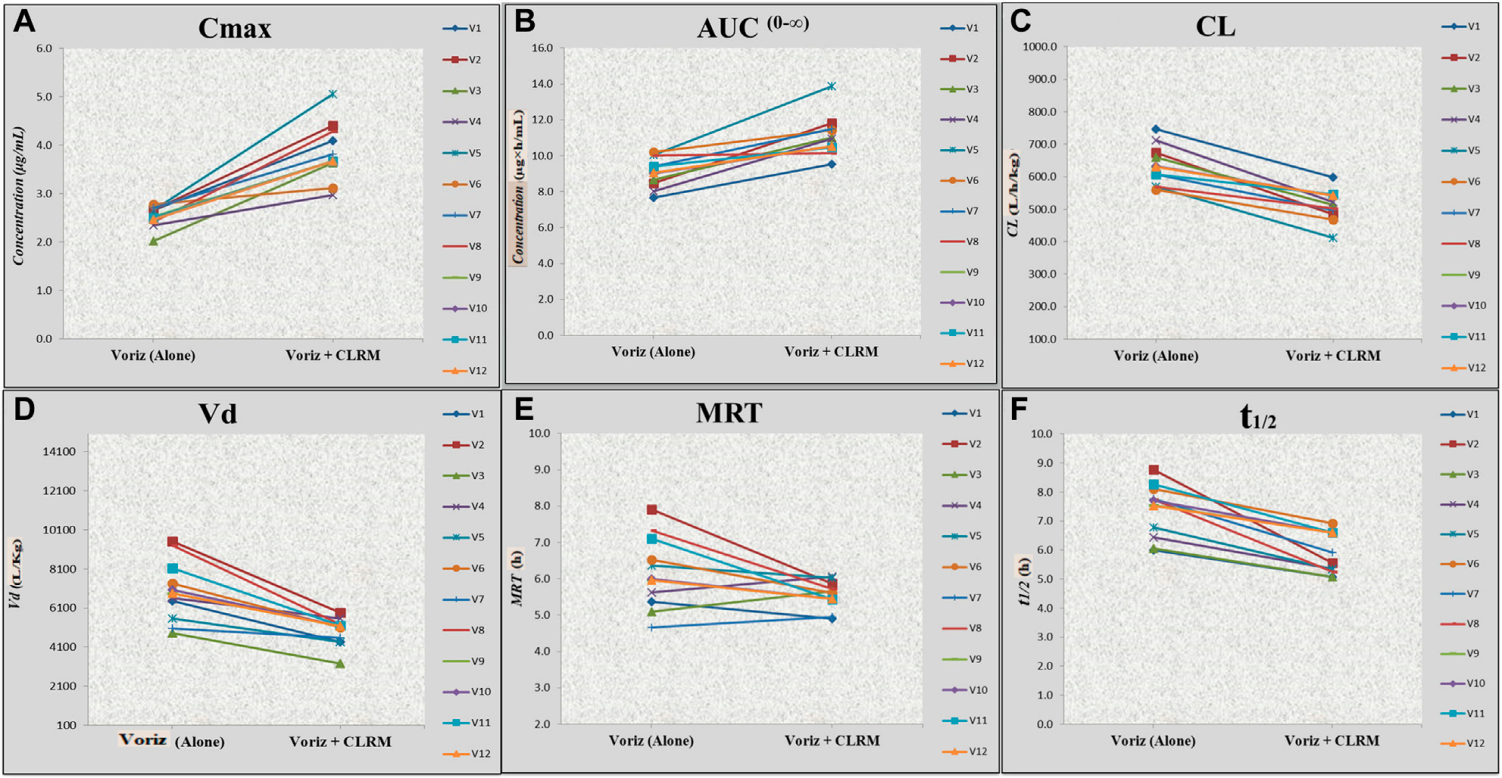
Effect of voriconazole alone and concurrent administration of voriconazole with clarithromycin on C_max_, AUC^0–∞^, MRT, apparent Cl, apparent Vd, and t_1/2_ of voriconazole in healthy volunteers. *Voriz, voriconazole; Voriz + CLRM, concurrent administration of voriconazole with clarithromycin; **(A)** Cmax, maximum plasma concentration; **(B)** AUC, area under curve; **(C)** Cl, apparent clearance; **(D)** Vd, apparent volume of distribution; **(E)** MRT, mean residence time; **(F)** t1/2, elimination half-life.

After co-administration of voriconazole 400 mg (200 mg × 2 mg tablets of voriconazole) with clarithromycin 500 mg tablet, a significant difference was observed in the C_max_ of voriconazole (Table 3). The geometric mean ratio of C_max_ for voriconazole was 1.52 (52% higher; 90% CI 1.04, 1.55; *p* = 0.000), which did not fall wholly within the acceptance region (0.80–1.25). Similarly, the geometric mean ratio of AUC^0−t^ and AUC^0-∞^ for voriconazole was 1.15 (16% higher; 90% CI 8.08, 10.02; *p* = 0.007) and 1.14 (21% higher; 90% CI 9.09, 10.02; *p* = 0.013), respectively, which fell wholly within the acceptance region (0.80–1.25). However, the geometric mean ratio of apparent Vd and apparent CL for voriconazole was 0.76 (23% decrease; 90% CI 5.00, 6.20; *p* = 0.051), and a 0.87 (13% decrease; 90% CI 41.95, 45.73; *p* = 0.019), respectively. Furthermore, the geometric mean ratio of E-t_1/2_ and MRT for voriconazole was 0.88 (26% decrease; 90% CI 1.88, 2.05; *p* = 0.371) and 0.89 (10% decrease; 90% CI 1.69, 1.82; *p* = 0.321), which fell wholly within the acceptance region (0.80–1.25). Likewise, the geometric mean ratio of E_rate_ for voriconazole was 1.14 (12% increase; 90% CI 0.09, 0.11; *p* = 0.44), which fell wholly within the acceptance region (0.80–1.25). The geometric mean ratio of all PK parameters of voriconazole fell within the acceptance region except C_max_ and Vd. In addition, there was a significant difference in t_max_ for voriconazole 25% decrease (2.00 ± 0 h to 1.50 ± 0 h). Further, the results are presented graphically in Figures 3A–F, representing individual data, whereas the mean data and the standard deviation have already been presented in Table 3.

## 4 Discussion

Antibiotics and antifungals are sometimes administered in conjunction in clinical therapeutic settings (Purkins et al., 2003a; Soler-Palacín et al., 2012; Mishima et al., 2017; Hirai et al., 2022). For instance, voriconazole and clarithromycin are prescribed simultaneously to treat Invasive Pulmonary Aspergillosis. It is a serious and often life-threatening fungal infection that commonly affects immune-compromised patients, such as those with hematologic malignancies or undergoing solid organ transplantation (Soler-Palacín et al., 2012). Voriconazole is considered a first-line treatment for Invasive Pulmonary Aspergillosis, and clarithromycin may be prescribed concomitantly to treat bacterial coinfections or to provide additional coverage against atypical bacteria (Purkins et al., 2003c; Soler-Palacín et al., 2012; Xing et al., 2017). Another indication that voriconazole and clarithromycin may be prescribed together is in treating nontuberculous mycobacterial infections, particularly those caused by *Mycobacterium Avium* Complex (MAC). Clarithromycin is often used as part of the multidrug regimen for MAC infections, and voriconazole may be added in cases where there is coexisting fungal infection or suspected fungal coinfection (Purkins et al., 2003c; Xing et al., 2017). Likewise, voriconazole and clarithromycin are prescribed simultaneously in treating infectious endophthalmitis (Purkins et al., 2003c; Mishima et al., 2017; Xing et al., 2017). Further, both participating drugs share the same enzyme pathway, providing a basis for evaluating the PK-DDI behavior of voriconazole and clarithromycin. Non-linear pharmacokinetic behavior of voriconazole is providing a base for many DDIs (Brüggemann et al., 2009). Voriconazole is a CYP2C19 and CYP3A4 enzyme inhibitor and a substrate of these enzymes (Mikus et al., 2011). Clarithromycin is a substrate and potent inhibitor of CYP3A4. It also moderately inhibits the CYP2C19 (Furuta et al., 1999), i.e., a main metabolic enzyme of voriconazole.

A significant increase in the C_max_ value (52%), and also AUC^0-∞^ value (21%) of voriconazole was observed after concomitant administration of clarithromycin, which is practically considered to be of clinical importance. The reason for this increment in AUC and C_max_ of voriconazole may be the inhibition of CYP3A4 and CYP2C19 enzymes by clarithromycin because clarithromycin is substrate and inhibitor of these enzymes (Furuta et al., 1999). Similar results were reported in many studies (Purkins et al., 2003b; Wood et al., 2003; Heinz et al., 2007; Andrews et al., 2008; Yasu et al., 2016) that demonstrated the effect of CYP2C19 and CYP3A4 inhibition has been evaluated over the PK of voriconazole and reported the increment of the AUC and C_max_ of voriconazole. Table 4 represents the increment in AUC and C_max_ of voriconazole due to inhibition of CYP isoenzyme by ethinyloestradiol and norethindrone (Andrews et al., 2008), cimetidine and ranitidine (Purkins et al., 2003b), omeprazole (Wood et al., 2003), pantoprazole (Heinz et al., 2007), lansoprazole (Yasu et al., 2016), esomeprazole (Bouatou et al., 2014), tacrolimus (Mochizuki et al., 2015), haloperidol (Motta et al., 2016), etravirine (Kakuda et al., 2013), azithromycin and erythromycin (Purkins et al., 2003d).

**TABLE 4:** Increased C_max_ and AUC of Voriconazole as Outcome of DDIs between Voriconazole and Interacting drug.

S. No.	Effector drug	*↑C_max_	*↑AUC	Reasons	Reference
1	Cimetidine	*↑18.5%	*↑22.5%	CYP450 enzyme inhibition	Purkins et al. (2003b)
2	Ranitidine	*↑3.5%	*↑4%	CYP450 enzyme inhibition	Purkins et al. (2003b)
3	Pantoprazole	**↑** ^NR*^	**↑** ^NR*^	Affinity to CYP isoenzymes	Heinz et al. (2007)
4	Esomeprazole	**↑** ^NR*^	**↑** ^NR*^	CYP2C19 inhibitor	Bouatou et al. (2014)
5	Lanoprazole	**↑** ^NR*^	**↑** ^NR*^	Low competitive inhibition for CYP2C19 and CYP3A4	Yasu et al. (2016)
6	Omeprazole	*↑15%	*↑41%	CYP2C19 and 3A4 inhibition	Wood et al. (2003)
7	Norethindrone ethinyloestradiol	*↑14%	*↑46%	CYP2C19 inhibition	Andrews et al. (2008)
8	Etravirine	*↑23%	*↑14%	CYP2C19 and 2C9 inhibition	Kakuda et al., (2013), Calcagno et al., (2014)
9	Haloperidol	**↑** ^NR*^	**↑** ^NR*^	weak CYP3A4 inhibition	Motta et al. (2016)
10	Erythromycin	*↑8%	*↑1%	CYP3A4 inhibition	Purkins et al. (2003d)
11	Azithromycin	*↑18%	*↑8%	CYP3A4 inhibition	Purkins et al. (2003d)

* NR, Not-reported (C_max_ and AUC, increased but not reported in exact percentage) *↑: Increased C_max_: Maximum plasma concentration AUC: area under curve.

A decrease in apparent clearance and an increase in AUC were observed in our study. This interaction may be possible by two mechanisms; decreased metabolism and interaction at the transporter level. *In-vitro* data suggested that clarithromycin inhibitory concentration of CYP34A is 48% and CYP2C19, as well as 2C9 values are 11% and 4%, respectively (Obach et al., 2006). The pattern of clarithromycin predicted a slighter decrease in the metabolism of the CYP2C19 substrate (Obach et al., 2005). Voriconazole has a greater affinity for CYP2C19, so it is expected that less increment in C_max_ of voriconazole should be the outcome because CYP34A is not a primary elimination pathway (Obach et al., 2005; Obach et al., 2006). In comparison, clarithromycin has a 60%–70% potential to inhibit the CYP3A4 at the intestinal level (Obach et al., 2006; Galetin et al., 2007). Therefore, clarithromycin potentially inhibited the metabolism of pimozide and midazolam (substrates for the CYP3A4 activity), as reported by several researchers (Gorski et al., 1998; Desta et al., 1999).

In addition, clarithromycin is a potent CYP3A4 inhibitor (inhibition constant $\left|Ki\right|$ = 57.5 µM; $K_{I}$ = 13.2 µM; $K_{inact}$ = 0.058/min) (Elsby et al., 2019). The Michaelis-Menten constant ( $K_{m}$ ) of voriconazole oxidase activity was 235 μM/L for CYP3A4 expressed in human CYP enzyme, which shows low affinity towards CYP3A4 (Mikus et al., 2006). Therefore, when the $K_{m}$ and $K_{i}$ values of these substances ( $K_{m}$ for CYP3A metabolism of voriconazole, 235 μM/L; $K_{i}$ for CYP3A4 inhibition by clarithromycin; $\left|Ki\right|$ = 57.5 µM) were taken into account, an interaction was expected with clarithromycin (Quinney et al., 2009; Burt et al., 2010).

Our results showed a 52% increase in plasma concentration might be because of decrease in the metabolism of voriconazole by clarithromycin. Nevertheless, the exact extent of DDI is not predictable because no *in-vivo* data show significant interaction (i.e., a significant increase in voriconazole plasma concentration) with another macrolide. The decrease in apparent clearance observed in our study may be because both interacting drugs are substrates and inhibitors of p-glycoprotein/ABCB1 transporter at intestinal, hepatic, and renal levels (Mikus et al., 2011; Müller and Fromm, 2011; Lempers et al., 2016). Clarithromycin has the potential to inhibit the various transporters because *in-vitro* data suggested that ${IC}_{50}$ values of clarithromycin for P-glycoprotein and MRP2 were 8.9 ± 0.5 µM and >50 μM, respectively (Vermeer et al., 2016). Interestingly, clarithromycin also has inhibitory potential against OATP transporter, e.g., ${IC}_{50}\;of$ OATP1B1 and 1B3 are 5.3 ± 1.3 µM and 14 ± 2 μM, respectively (Müller and Fromm, 2011; Vermeer et al., 2016).

The equation $t_{1/2}=\frac{0.693}{Erate}$ shows that half-life is inversely proportional to the elimination rate constant. Our study results show an increase in elimination rate constant (E_rate_) from 0.09/hour to 0.11/hour, so half-life became reduced. It may be due to transporter involvement (Mikus et al., 2011; Müller and Fromm, 2011; Peters et al., 2011; Lempers et al., 2016; Allegra et al., 2018). The reduction in half-life could be due to changes in the elimination rate constant. Merely looking into the overlay graph of voriconazole alone and voriconazole concentration after administration of voriconazole along clarithromycin depicted that initially faster elimination rate and decreased in half-life, later on, elimination became slow, so overall decreased in the apparent clearance of voriconazole has been observed.

According to the equation, i.e., $t_{1/2}=\frac{0.693\;x\;Vd}{Cl}$ , a decrease in apparent clearance should generally increase the half-life. However, our results showed a decrease in the half-life of voriconazole. Similar results have been presented by Rengelshausen et al. (Rengelshausen et al., 2005), who have evaluated the impact of concomitantly administrated voriconazole with St. John’swort. They have observed a 20% reduction in the half-life of voriconazole. They suggested an increase in oral BA, a reduction in the distribution of voriconazole, and a short-term decrease of the systemic voriconazole distribution may be due to alteration in the transport process, and these are the probable mechanism of the reduction in the half-life of voriconazole besides decreased apparent clearance (Rengelshausen et al., 2005).

Our results showed a decrease in the apparent volume of distribution. According to the apparent clearance equation, i.e., Cl = KVd, when Vd decreases that leads to a decreased clearance value, provided that the elimination rate constant remains the same. A reduction in apparent clearance has been observed in our results. Wakasugi et al. (Wakasugi et al., 1998) have reported an increase in the AUC and C_max_ of digoxin on the concomitant administration of clarithromycin with digoxin by inhibiting p-glycoprotein (Wakasugi et al., 1998). Clarithromycin may reduce the voriconazole apparent clearance by competition and inhibiting the P-glycoprotein transporter. At the hepatocyte level, voriconazole and clarithromycin interaction may be possible because clarithromycin is an inhibitor of the SLCO1B3 (OATP1B3) transporter (Müller and Fromm, 2011), and voriconazole is a substrate of this transporter. An increase in AUC and C_max_ and reduced voriconazole apparent clearance may be due to the inhibition of hepatocellular uptake transporters (SLCO1B3/OATP1B3). Consequently, a reduced hepatic influx of voriconazole may lead to a reduction in metabolism (Allegra et al., 2018). A similar mechanism of DDI was presented between clarithromycin and paclitaxel involving hepatic OATP1B3 transporter inhibition. Efflux transporter, i.e., ABCC2, also known as MRP2, is common transporter for both interacting drugs (Peters et al., 2011; Allegra et al., 2018). Therefore, PK-DDIs may be possible among voriconazole and clarithromycin for competition for that common transporter. Interestingly, both interacting drugs (clarithromycin and voriconazole) are inhibitors of this transporter (Peters et al., 2011; Allegra et al., 2018).

A sharp difference in t_max_ from 2 h to 1.5 h (which means a 25% decrease) has been observed in our study. The decline in t_max_ may be due to the physiochemical nature of both interacting drugs. According to Biopharmaceutics Classification System (BCS), voriconazole (Kumar et al., 2014) and clarithromycin (Kristin et al., 2017) are both class-II drugs. Clarithromycin is a weak base with 8.87 pKa (Nakagawa et al., 1992; Grübel and Cave, 1998). Voriconazole exhibits basic and acidic pKa profiles, i.e., basic pKa: 1.76 (Adams and Bergold, 2005; Adams et al., 2008) and acidic pKa: 12.7 and 4.36 (Owens et al., 2000; Damle et al., 2011; Vanstraelen et al., 2015). Voriconazole nature may be a cause of this interaction. Clarithromycin is basic in nature and may provide a medium for the solubility of an acidic moiety of voriconazole. It is possible that acidic pKa is predominant at this stage, which is why t_max_ decreased and enhanced the dissolution. Likewise, clarithromycin is also a potent inhibitor of efflux transporter, i.e., p-glycoprotein (Müller and Fromm, 2011) and CYP450 enzyme system (CYP3A4 and CYP2C19) at the intestinal level (Furuta et al., 1999). So, these two reasons enhanced the absorption rate and decreased the t_max_ of voriconazole. Similar results have been reported by Rengelshausen et al., which demonstrated that St. John’swort decreased the t_max_ of voriconazole due to enhancing the dissolution rate (Rengelshausen et al., 2005). However, the present study was a single-dose study and single dose might not enhance the dissolution; further investigations are required to evaluate the PK profile of voriconazole in case of multiple dosing and a larger population.

### 4.1 Recommendation

The PK-DDI study among voriconazole and clarithromycin has demonstrated the alteration in the PK parameters of voriconazole. We observed that the C_max_ of voriconazole has significantly altered in this interaction. Therefore, adjustments in dosage regimens of voriconazole are required. Also, therapeutic drug monitoring (TDM) is necessary while administering clarithromycin along with voriconazole at the usual recommended doses (200–400 mg). In long-term therapy, dose adjustments may be required because the voriconazole therapeutic range is narrow (Ashbee et al., 2013). Therefore, the chances of toxicity are enhanced, so monitoring should be required for plasma voriconazole concentration. Then a reduction in the dose shall be opted for according to the patient’s condition. If it is not workable, prescribing an alternative is the best option. Another drug of the macrolide family, such as erythromycin, has a non-significant effect on the PK parameters of voriconazole (Purkins et al., 2003d). Therefore, erythromycin can be effectively administered instead of clarithromycin.

### 4.2 Limitations and future perspective

The present study was a single-dose study; further investigations are required to evaluate the PK profile of voriconazole in case of multiple dosing and a larger population. Furthermore, voriconazole mainly metabolizes from CYP2C19, and the 2C19 enzyme has polymorphism. Therefore, a research study is also required to enlighten the impact of CYP2C19 genotyping/phenotyping on the PK parameters of voriconazole in Pakistani populations. In addition, the pharmacokinetics of voriconazole in pediatric patients differ from adults, with reduced oral bioavailability potentially due to greater systemic and first-pass metabolism in children. Clearance rates may also vary among different genotypes in pediatric patients compared to adults, potentially influenced by limited data availability for certain genotypes (Karlsson et al., 2009; Wu et al., 2022). One study revealed a high incidence of clinically significant QTc prolongation in pediatric patients treated with voriconazole. Therefore, vigilant monitoring of QTc interval, along with laboratory assessments and correction of electrolyte imbalances, is crucial in order to prevent cardiac arrhythmias in this vulnerable patient population (Pasternak et al., 2019). Therapeutic drug monitoring (TDM) of voriconazole is necessary to individualize dosing regimens in pediatric oncology patients, as optimal doses vary widely in this population. Younger patients may be at higher risk for poor outcomes and may require additional monitoring and dose adjustment. Further research with larger sample sizes and comprehensive pharmacokinetic data is needed to better understand the impact of age and genotype on voriconazole pharmacokinetics in pediatric patients and optimize dosing strategies for improved patient outcomes (Walsh et al., 2010; Liu and Mould, 2014; Tucker et al., 2015). Nevertheless, our current study presented a significant PK-DDI between voriconazole and clarithromycin. Indeed, which will be helpful for all healthcare providers regarding the safe and effective therapy of voriconazole.

## 5 Conclusion

A clinically significant PK-DDI of voriconazole and clarithromycin has been observed. In addition, we observed a 52% increase in the C_max_ of voriconazole during the co-administration of clarithromycin with voriconazole. Therefore, the dose of voriconazole must be adjusted to avoid severe and dangerous side effects like hepatotoxicity and neurotoxicity because voriconazole is a narrow therapeutic index drug.

## Data Availability

The datasets presented in this study can be found in online repositories. The names of the repository/repositories and accession number(s) can be found in the article/supplementary material.

## Abbreviations

CLRM, clarithromycin; DDI’s, parameters, drug-drug interactions; HPLC, high-performance-liquid chromatography; PK, pharmacokinetic; PK-DDI, pharmacokinetic drug-drug interaction; RP/HPLC, reversed-phase/HPLC; Voriz, voriconazole.
